# Adjustment of the GRACE score by HemoglobinA1c enables a more accurate prediction of long-term major adverse cardiac events in acute coronary syndrome without diabetes undergoing percutaneous coronary intervention

**DOI:** 10.1186/s12933-015-0274-4

**Published:** 2015-08-19

**Authors:** Xiao-Jun Liu, Zhao-Fei Wan, Na Zhao, Ya-Ping Zhang, Lan Mi, Xin-Hong Wang, Dong Zhou, Yan Wu, Zu-Yi Yuan

**Affiliations:** Department of Cardiovascular Medicine, First Affiliated Hospital of Xi’an Jiaotong University, Xi’an, 710061 Shaanxi China; First Department of Cardiology, Shaanxi Provincial People’s Hospital, Xi’an, Shaanxi China; Department of Cardiovascular Medicine, Second Affiliated Hospital of Xi’an Jiaotong University, Xi’an, Shaanxi China; Key Laboratory of Environment and Genes Related to Diseases, Xi’an Jiaotong University, Ministry of Education, Xi’an, Shaanxi China; Department of Ophthalmology Medicine, Xi’an IV People’s Hospital, Xi’an, Shaanxi China; Peking University Cancer Hospital and Institute, Beijing, China

## Abstract

**Background:**

The Global Registry of Acute Coronary Events (GRACE) risk score is widely recommended for risk assessment in patients with acute coronary syndrome (ACS). Chronic hyperglycemia [hemoglobinA1c (HbA1c)] can independently predict major adverse cardiac events (MACEs) in patients with ACS. We investigated whether the prediction of MACEs with the GRACE score could be improved with the addition of HbA1c content in ACS patients without diabetes mellitus (DM) undergoing percutaneous coronary intervention (PCI).

**Methods:**

We enrolled 549 ACS patients without DM who underwent PCI. The GRACE score and HbA1c content were determined on admission. Correlation was analyzed by Spearman’s rank correlation. Cumulative MACE curve was calculated using the Kaplan–Meier method. Multivariate Cox regression was used to identify predictors of MACEs. Additionally, the predictive value of HbA1c content alone and combined with GRACE score was estimated by the area under the receiver-operating characteristic curve (AUC), continuous net reclassification improvement (NRI) and integrated discrimination improvement (IDI).

**Results:**

During a median of 42.3 months (interquartile range 39.3–44.2 months), 16 (2.9 %) were lost to follow-up, and patients experienced 69 (12.9 %) MACEs: 51 (9.6 %) all-cause deaths and 18 (3.4 %) nonfatal myocardial infarction cases. The GRACE score was positively associated with HbA1c content. Multivariate Cox analysis showed that both GRACE score and HbA1c content were independent predictors of MACEs (hazard ratio 1.030; 95 % CI 1.020–1.040; *p* < 0.001; 3.530; 95 % CI 1.927–6.466; *p* < 0.001, respectively). Furthermore, Kaplan–Meier analysis demonstrated increased risk of MACEs with increasing HbA1c content (log-rank 33.906, *p* < 0.001). Adjustment of the GRACE risk estimate by HbA1c improved the predictive value of the GRACE score [increase in AUC from 0.75 for the GRACE score to 0.80 for the GRACE score plus HbA1c, *p* = 0.012; IDI = 0.055, *p* < 0.001; NRI (>0) = 0.70, *p* < 0.001].

**Conclusions:**

HbA1c content is positively associated with GRACE risk score and their combination further improved the risk stratification for ACS patients without DM undergoing PCI.

**Electronic supplementary material:**

The online version of this article (doi:10.1186/s12933-015-0274-4) contains supplementary material, which is available to authorized users.

## Background

Patients with acute coronary syndrome (ACS) are diverse in terms of clinical presentation and risk of death or disability. Accurate management decisions with comprehensive evaluation may improve the outcomes of patients at high risk. To identify high-risk patients, current guidelines recommend a standardized approach involving validated scoring systems such as the Global Registry of Acute Coronary Events (GRACE) score [1–3]. Although the GRACE risk score has been validated, the score does not include measurement of important biomarkers. Therefore, whether combining other biomarkers with the GRACE score can provide a more accurate risk estimation in ACS needs to be explored.

Long-term glycometabolic disorder implies high risk for cardiovascular disease [4, 5]. Glycosylated hemoglobin (HbA1c) is a well-known biomarker of long-term glycometabolic state and is minimally affected by stress during ACS. Previous research found elevated HbA1c content related to increased risk of cardiovascular events [6–8].

In the present study, we investigated the predictive value of HbA1c content and GRACE score individually for major adverse cardiac events (MACEs) in patients with ACS but without diabetes mellitus (DM) undergoing PCI and the potential incremental prognostic value of HbA1c content added to GRACE score.

## Methods

### Study cohort

We performed a single-center, observational study of consecutive non-diabetes patients with ACS performed PCI in the First Affiliated Hospital of Medical College of Xi’an Jiaotong University from December 2010 to December 2011, which included unstable angina, non-ST-segment elevation myocardial infarction (NSTEMI), and ST-segment elevation MI (STEMI).

They all performed PCI using standard techniques after Qualitative and quantitative coronary angiographic analyses. All procedural decisions, including device selection and adjunctive pharmacotherapy, were made at the discretion of experienced interventional cardiologists according to 2007 focused update of the ACC/AHA/SCAI 2005 guideline update for percutaneous coronary intervention [9]. The diagnostic criteria of DM were: HbA1c ≥6.5 %, FPG ≥7.0 mmol/L, 2-h PG ≥11.1 mmol/L according to 2010 ADA Diagnosis and classification of diabetes mellitus [10].

Exclusion criteria were history of DM, treatment with diabetes drugs, HbA1c content ≥6.5 % on admission, no treatment with PCI, advanced liver disease, renal failure, cancer, valvular heart disease, stroke, idiopathic dilated or hypertrophic cardiomyopathy, peripheral arterial disease, pregnancy, receiving anti-inflammatory drugs, acute or chronic infections or autoimmune disease, and blood or thyroid disease.

The study complied with the Declaration of Helsinki and was approved by the ethics committee of the hospital. Written informed consent was obtained from all patients.

### Demographic and clinical data

Main demographic data, cardiovascular risk factors and cardiovascular drugs received were obtained from medical records. Current smokers were defined as having smoked more than 100 cigarettes during their lifetime and still smoking in the past 30 days. Hypertension was defined as resting blood pressure ≥140/90 mmHg at two different visits or receiving hypertension drugs. Previous MI was based on a history of acute MI (AMI) or with signs of an infarction outside the area of the index infarction.

### Blood samples

Peripheral blood was sampled from patients in a fasting state the morning following the admission day. Venous plasma concentrations of glucose, lipids, lipoproteins, serum creatinine, N-terminal pro-B-type natriuretic peptide (NT-proBNP), white blood cell, platelet count (PLT), Neutrophile count, Monocyte count and HbA1c content (normal values 4–6 %) were determined in the biochemistry department using standard biochemical techniques for the hospital. We calculated estimated glomerular filtration rate as (ml min^−1^ 1.73 m^−2^) = 194 × (serum creatinine)^−1.094^ × (age)^−0.287^(× 0.739 if female).

They all performed Echocardiography for left ventricle function through left ventricle ejection fraction (LVEF).

### Calculation of GRACE risk score

The GRACE risk prediction tool was previously described [11]. The score is derived from several variables (age, heart rate, systolic blood pressure, creatinine level, congestive heart failure, in-hospital percutaneous coronary intervention, in-hospital coronary aortic bypass grafting, history of MI, ST-segment depression, and elevated cardiac enzyme/marker levels) and calculated for each patient. The GRACE risk score was originally designed to predict post-discharge 6 month mortality and had been shown to provide good discrimination of mortality up to 4 years after an ischemic event [12–14].

### Outcomes and follow-up

All-cause death and nonfatal MI were defined as MACEs. All patients were followed up by interview or telephone in our hospital, and the end of follow-up was the date of the first MACE occurrence obtained by reviewing hospital records. Some patients were followed up until December 2014.

### Statistical analysis

Data were analyzed by use of SPSS 19.0 for Windows (SPSS Inc., Chicago, IL, USA). Continuous variables were expressed as mean ± SD. Categorical variables were expressed as frequency and percentage. The Kolmogorov–Smirnov test was used to assess normal distribution of quantitative variables. Independent samples *t* test was used to compare two groups, and categorical variables were compared by Chi square test. One-way ANOVA was used to compare multiple groups. To limit the influence of extreme observations, NT-proBNP was natural logarithmically transformed to obtain Ln NT-proBNP. Correlation was analyzed by Spearman’s rank correlation. Univariate and multivariate survival analyses involved Cox regression analysis. To assess the prognostic value of HbA1c content, Kaplan–Meier survival curves were used. Additionally, the incremental predictive value resulting from adding HbA1c variable to GRACE risk score was analyzed in the validation set using several measures of improvement in discrimination: increase in the area under the receiver-operating characteristic (ROC) curve (AUC), as well as integrated discrimination improvement (IDI), and continuous net reclassification improvement (NRI). DeLong’s test was used to compare the AUC from each of models [15], which were analyzed by use of MedCalc Version 11.4.2.0. The IDI was equal to the increase in discrimination slope defined as the mean difference in predicted risks between those with and without events. The continuous NRI was a non-parametric analogue of the IDI and equals twice the difference in probabilities of upward reclassification for events minus for non-events [16], which were analyzed by use of SAS 9.2 (SAS Institute Inc., Cary, NC, USA).

All probability values were two-tailed. *P* < 0.05 was considered statistically significant.

## Results

### Baseline characteristics of patients

During a median of 42.3 months (interquartile range 39.3–44.2 months), 16 (2.9 %) were lost to follow-up. In the study, a total of 533 consecutive patients (a mean age of 59.96 ± 12.65 years, 67.7 % man) included 203 unstable angina, 95 NSTEMI and 235 STEMI, the HbA1c content in each type of ACS was respectively 5.588 ± 0.45 %, 5.598 ± 0.51 %, 5.610 ± 0.45 %, in which there was no statistical differences (*p* = 0.878). Among the 533 patients, 69 (12.9 %) experienced a MACE, including 51 (9.6 %) all-cause deaths and 18 (3.4 %) nonfatal myocardial infarction cases. The event rate of each type of ACS was 9.85 % (20/203), 15.78 % (15/95), 14.46 % (34/235). All patients were segregated into three groups by tertiles of baseline HbA1c content (≤5.4 %, 5.5–5.8 %, 5.9–6.4 %). Baseline characteristics were shown in Additional file 1: Table S1: Patients in the higher HbA1c levels more often had a prior history of PCI, were lower ejection fraction, eGFR level, as well as higher in Monocyte count and LnNT-ProBNP.

### Clinical characteristics of patients with and without MACEs

Patients with MACEs were elderly, had more frequent prior history of hypertension, prior history of MI, prior history of PCI and were higher LnNT-ProBNP, PLT, monocyte count, GRACE score and HbA1c content as well as lower ejection fraction compared to patients without MACEs (Table 1). We carried out the correlation analysis between the GRACE risk score and HbA1c levels as continuous variables and showed that GRACE score was positively correlated with HbA1c content (R = 0.192, *p* < 0.001).

**Table 1:** Characteristics of non-DM patients with ACS undergoing PCI with or without major adverse cardiac events (MACEs)

Variable	All patients (n = 533)	With MACE (n = 69)	Without MACE (n = 464)	*p* value
Age (year)	59.96 ± 12.65	64.58 ± 11.43	59.27 ± 12.69	*0.001*
Sex				
Male	361 (67.7)	47 (68.1)	314 (67.7)	0.941
BMI (kg/m^2^)	24.11 ± 2.70	24.60 ± 2.96	24.04 ± 2.65	0.111
Hypertension	195 (36.6)	40 (58.0)	155 (33.4)	*<0.001*
Smoking	333 (62.5)	48 (69.6)	285 (61.4)	0.192
Prior MI	37 (6.9)	13 (18.8)	24 (5.2)	*<0.001*
Prior PCI	24 (4.5)	7 (10.1)	17(3.7)	*0.015*
DBP (mmHg)	77.94 ± 13.00	77.88 ± 12.40	77.95 ± 13.11	0.967
SBP (mmHg)	125.53 ± 20.02	126.35 ± 21.78	125.41 ± 19.77	0.716
Heart rate (bpm)	75.36 ± 13.27	77.12 ± 13.50	75.10 ± 13.24	0.240
eGFR (mL min^−1^ 1.73 m^−2^)	88.60 ± 35.50	87.05 ± 37.31	88.83 ± 35.26	0.697
FBS (mmol/L)	6.37 ± 1.82	6.28 ± 1.35	6.39 ± 1.88	0.652
TC (mmol/L)	3.92 ± 1.08	3.94 ± 0.92	3.92 ± 1.10	0.926
TG (mmol/L)	1.62 ± 1.00	1.47 ± 1.07	1.65 ± 0.99	0.171
HDL (mmol/L)	1.02 ± 0.25	1.04 ± 0.26	1.02 ± 0.25	0.541
LDL (mmol/L)	2.28 ± 0.74	2.30 ± 0.75	2.27 ± 0.74	0.774
Apo A1 (g/L)	1.08 ± 0.18	1.08 ± 0.22	1. 08 ± 0.18	0.894
Apo B (g/L)	0.77 ± 0.23	0.76 ± 0.22	0.77 ± 0.23	0.904
LVEF (%)	54.26 ± 12.34	49.68 ± 12.23	54.94 ± 12.15	*0.001*
NT-proBNP (pg/mL)	5.86 ± 1.60	6.30 ± 1.82	5.79 ± 1.56	*0.013*
PLT count (10^9^/L)	192.81 ± 68.17	212.67 ± 82.73	189.86 ± 65.32	*0.009*
WBC count (10^9^/L)	8.05 ± 3.23	8.38 ± 3.56	8.00 ± 3.18	0.366
Monocyte count (10^9^/L)	0.57 ± 0.32	0.65 ± 0.45	0.55 ± 0.30	*0.021*
Neutrophile count (10^9^/L)	5.92 ± 3.14	6.36 ± 3.21	5.85 ± 3.13	0.213
HbA1c content (%)	5.60 ± 0.46	5.90 ± 0.33	5.56 ± 0.46	*<0.001*
GRACE score	110.47 ± 27.13	131.45 ± 24.40	107.35 ± 26.13	*<0.001*
Medication at discharge				
Aspirin	524 (98.3)	66 (95.7)	458 (98.7)	0.066
Clopidogrel	527 (98.9)	68 (98.5)	459 (98.9)	0.785
Statins	430 (80.7)	58 (84.1)	372 (80.2)	0.446
ACEI/ARB	389 (73.0)	46 (66.7)	343 (73.9)	0.205
β-blockers	347 (65.1)	42 (60.9)	305 (65.7)	0.429

Data are mean ± SD or n (%)

*BMI* body mass index, *Prior MI* prior myocardial infarction, *Prior PCI* prior percutaneous coronary intervention, *SBP* systolic blood pressure, *DBP* diastolic blood pressure, *eGFR* estimated glomerular filtration rate, *FBS* fasting blood sugar, *TC* total cholesterol, *TG* triglycerides, *HDL-C* high-density lipoprotein cholesterol, *LDL-C* low-density lipoprotein cholesterol, *Apo-A1* apolipoprotein A1, *Apo-B* apolipoprotein B, *NT-proBNP* N-terminal pro-B-type natriuretic peptide, *PLT* platelets, *LVEF* left ventricle ejection fraction, *WBC* white blood cell count, *ACEI* angiotensin-converting enzyme inhibition, *ARB* angiotensin receptor blocker, *HbA1c* hemoglobin A1c, *GRACE score* Global Registry of Acute Coronary Events (GRACE) score

### HbA1c content as an independent predictor of MACE occurrence

**Fig. 1:**
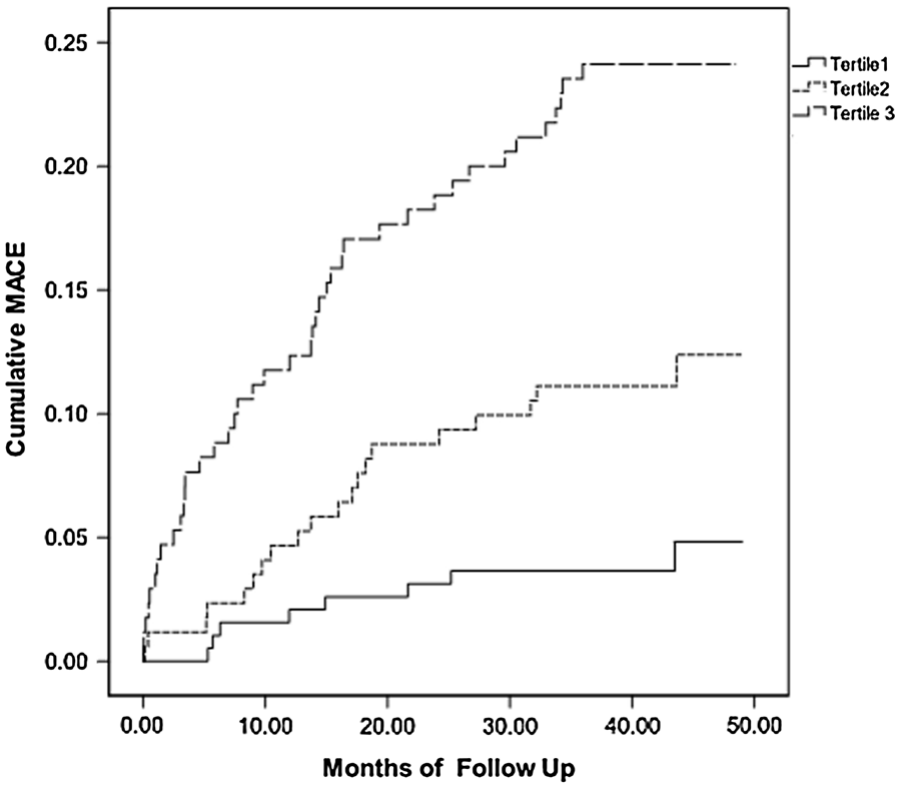
Kaplan–Meier analysis of major adverse cardiac events (MACEs) based on hemoglobin A1c (HbA1c) content. The 533 patients were divided by tertiles of HbA1c content: ≤5.4 %, 5.5–5.8 %, and 5.9–6.4 %. Risk of a MACE increased with increasing tertile of HbA1c content (log-rank test 33.906, *p* < 0.001)

On univariate Cox analysis, significant predictors of a MACE were age, hypertension, prior MI,prior PCI, LVEF, LnNT-ProBNP, PLT, monocyte count, HbA1c content and GRACE score (Table 2). On multivariate Cox analysis, HbA1c content was a significant and independent predictor of a MACE [hazard ratio (HR) 3.530; 95 % confidence interval (95 % CI) 1.927–6.466; *p* < 0.001] as was GRACE score (HR 1.030; 95 % CI 1.020–1.0403; *p* < 0.001) (Table 3). The cumulative risk of a MACE generally increased with elevated HbA1c content by Kaplan–Meier analysis (log-rank 33.906, *p* < 0.001; Fig. 1).

**Table 2:** Univariate Cox analysis for MACEs

Variable	HR	95 % CI	*p* value
Age (per 1 year)	1.032	1.012–1.052	*0.001*
Male (vs. female)	0.910	0.480–1.725	0.772
BMI (per 1 kg/m^2^)	1.071	0.984–1.166	0.111
Hypertension	2.571	1.594–4.148	*<0.001*
Smoking	1.438	0.861–2.402	0.165
Prior MI	3.454	1.888–6.319	*<0.001*
Prior PCI	2.839	1.299–6.206	*0.009*
DBP (per 1 mmHg)	1.000	0.982–1.018	0.976
SBP (per 1 mmHg)	1.002	0.991–1.014	0.716
Heart rate (per 1 mmHg)	1.010	0.993–1.026	0.240
eGFR (per 1 mL min^−1^ 1.73 m^−2^)	0.998	0.992–1.005	0.647
FBS (per 1 mmol/L)	0.976	0.856–1.112	0.712
TC (per 1 mmol/L)	1.013	0.820–1.252	0.903
TG (per 1 mmol/L)	0.787	0.566–1.094	0.153
HDL (per 1 mmol/L)	1.355	0.543–3.380	0.515
LDL (per 1 mmol/L)	1.048	0.767–1.432	0.766
Apo A1 (per 1 g/L)	0.926	0.248–3.451	0.908
Apo B (per 1 g/L)	0.955	0.338–2.697	0.931
LVEF (per 1 %)	0.965	0.945–0.985	*0.001*
NT-proBNP (per 1 ln unit)	1.236	1.052–1.451	*0.010*
PLT count (per 10^9^/L)	1.004	1.001–1.006	*0.006*
WBC count (per 10^9^/L)	1.035	0.966–1.109	0.326
Monocyte count (per 10^9^/L)	2.141	1.189–3.855	*0.011*
Neutrophile count (per 10^9^/L)	1.046	0.976–1.120	0.202
HbA1c content (per 1 %)	5.342	2.968–9.613	*<0.001*
GRACE score (per 1)	1.033	1.024–1.043	*<0.001*

*HR* hazard ratio, *95* *% CI* 95 % confidence interval

**Table 3:** Multivariate Cox analysis for MACEs

Variable	HR	95 % CI	*p* value
HbA1c (per 1 %)	3.530	1.927–6.466	<0.001
GRACE score (per 1)	1.030	1.020–1.040	<0.001
Hypertension	1.932	1.185–3.148	0.008
Prior MI	2.372	1.284–4.328	0.006
LVEF (per 1 %)	0.972	0.952–0.992	0.006
PLT (per 10^9^/L)	1.116	1.023–1.218	0.013

*HR* hazard ratio, *95* *% CI* 95 % confidence interval

### Effect of HbA1c content and GRACE score combined on MACE occurrence

**Fig. 2:**
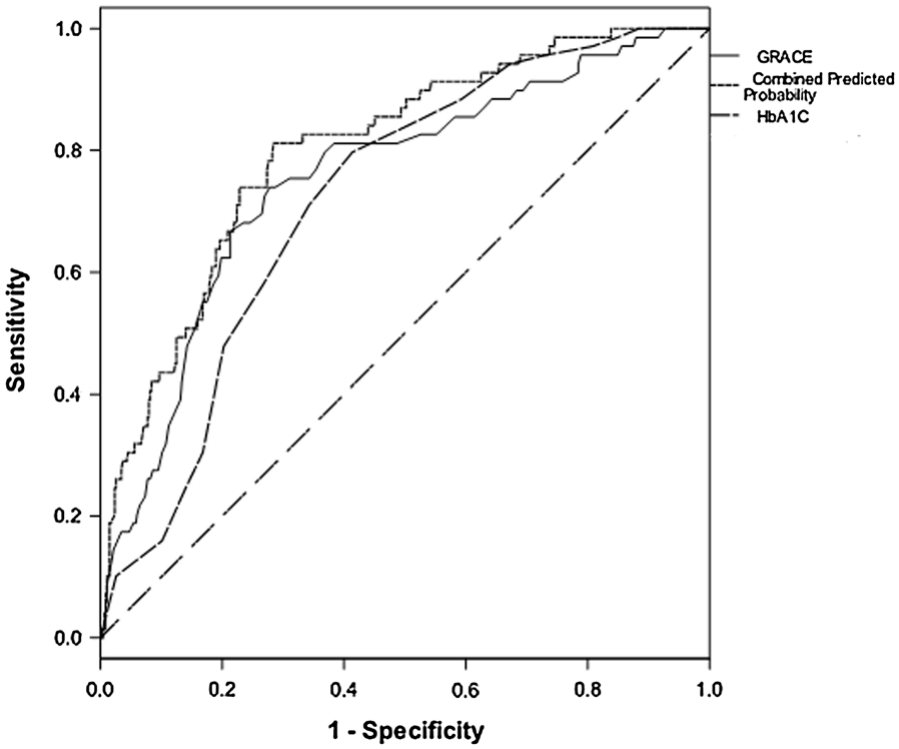
Receiver operating characteristic (ROC) curve analysis. The addition of HbA1c content to the GRACE score as continuous variables could improve the predictive power for long-term MACEs (area under the ROC curve for GRACE score alone, 0.75; combined with HbA1c content, 0.80; z value 2.521, *p* = 0.012)

Since both HbA1c content and GRACE score were independent risk factors of a MACE, we assessed the effect of their combination on predicting long-term risk of MACE occurrence. The AUC increased from 0.75 (95 % CI 0.69–0.82) for GRACE score alone to 0.80 (95 % CI 0.75–0.85) for GRACE score adjustment by HbA1c content (difference in the AUCs, 0.05; z value 2.521, *p* = 0.012) (Fig. 2). Addition of HbA1c content improved GRACE score alone model discrimination, which was confirmed by the IDI and the continuous, category-free NRI (>0). The IDI for HbA1c content was 0.055 (95 % CI 0.035–0.075, *P* < 0.001), suggesting further average separation of events from non-events by the HbA1c; the NRI (>0) for HbA1c content was 0.70, (95 % CI 0.47–0.94, *P* < 0.001), with events contributing 0.42 and non-events 0.28 (Table 4), showing that the HbA1c content led to a significant net reclassification of patients^,^ risk in the appropriate directions.

**Table 4:** Statistics for model improvement with the addition of HbA1c content

		*p* value
Events, n (%)	69 (12.9)	
Nonevents, n (%)	464 (87.1)	
Continuous NRI (%)		
cNRI_event_	42	
cNRI_nonevent_	28	
cNRI	70 (95 % CI 47–94)	*<0.001*
IDI statistics		
IDI	0.055 (95 % CI 0.035–0.075)	*<0.001*
AUC		
GRACE risk score	0.75 (95 % CI 0.69–0.82)	
GRACE + HbA1c	0.80 (95 % CI 0.75–0.85)	
Difference	0.05	*0.012*

*95* *% CI* 95 % confidence interval, *IDI* integrated discrimination improvement, *NRI* net reclassification improvement, *cNRI* continuous net reclassification improvement

## Discussion

Management decisions in ACS should be based on risk stratification of patients. The GRACE score provides validated prognostic information for MACEs in ACS patients [12, 14]. In accordance with previous research results, the GRACE score could independently predict a MACE in our ACS patients without DM undergoing PCI. However, the AUC with the GRACE score alone was only 0.75, which might be due to some potential risk factors not fully captured by the scoring system. Growing epidemiological evidence supports that HbA1c content in the general population and in patients with and without diabetes is an independent risk factor of cardiovascular events including MI [6, 8, 17]. However, HbA1c content has not been considered with the GRACE scoring system in previous research. In our patients, the GRACE score increased with increasing HbA1c content at baseline, and the 2 variables were correlated. Moreover, the GRACE score adjustment by HbA1c content on admission enhanced the predictive value for ACS patients without DM undergoing PCI (AUC increased from 0.75 for GRACE score alone to 0.80 for GRACE score plus HbA1c).To better assess the improvement in discrimination of GRACE score adjustment by HbA1c variable, we used new statistical metrics [IDI and a category-free, continuous NRI (>0)], and IDI for HbA1c showed further average separation of events from non-events by the HbA1c; Using the a category-free, continuous NRI (>0),we fund that a net 28 % of the patients without events were reclassified into lower risk and that a net 42 % of patients with events were reclassified into higher risk. The category-free, continuous NRI (>0) thus reached an impressive 0.70, which suggested that the HbA1c content led to a significant net reclassification of patients^,^ risk in the appropriate directions.

In the present study, the long-term event rate seems low compared previous researches. It may attribute to followed reasons. Firstly, the enrolled patients were non-DM, which were lower in the cardiovascular events rate than that in patients with diabetic mellitus, especially in the patients of poor nocturnal glycemic control [18]. Secondly, Recent some reports have shown ethnic differences effect the MACE occurrence rate after PCI not only between African Americans and whites [19, 20],but also in Asian subpopulations, such as compared to Indian, Chinese patients have lower Major Adverse Cardiovascular Events [21–23]. In addition, our results was comparable compared with the MACE rate documented by Clinical Pathways for Acute Coronary Syndromes in China (CPACS) study [24, 25].

Although some research had found that higher level of admission glucose predict a worse prognosis including mortality and MI in ACS patients [26–28], recent studies showed that the prediction value of admission glucose was not improved by combining GRACE score [29]. As different from admission glucose, HbA1c is an indicator of general glycometabolic state and is minimally affected by acute stress and also acute glucose management. Increased HbA1c content was not only showed as a indictor of complications including ACS [30] but also a predictor of long-term survival in ACS patients with and without DM [31–33]. Whether the HbA1c content in ACS patients without DM undergoing PCI could be an independent predictor of cardiovascular events is uncertain. In our study, HbA1c content at baseline was higher in patients with than without a MACE, and HbA1c content could independently predict a long-term MACE in ACS patients without DM undergoing PCI. Furthermore, we found that the predictive value on MACE of adding HbA1c on the top of GRACE score system.

Researches have found that C-reactive protein [34], NT-proBNP [35], erythrocyte fatty acids [36], growth differentiation factor-15 [37], cystatin C [38], dikkopf [39], RDW/PDW [40], high-sensitivity troponin [41], mean platelet volume(MPV) [42] and other factors add value to a scoring system for predicting adverse cardiovascular events after ACS. We found that GRACE score and baseline HbA1c content were positively correlated, and their combination improved the predictive value. Whether this combination can improve the outcome of ACS patients with DM needs further comprehensive investigation in clinical practice.

## Limitations

The number of patients in the cohort was relatively small. Therefore, these findings need to be verified by multicenter and larger cohort studies. In addition, this study was limited to Chinese subjects, so conclusions for other ethnic groups are cautioned.

## Conclusions

HbA1c content measured in ACS patients without DM undergoing PCI could predict a MACE and was positively related with GRACE score. The combination of the two factors may improve on risk stratification of ACS patients without DM undergoing PCI.

## Additional file

Additional file 1:
**Table S1.** Baseline characteristics of 533 non-diabetes mellitus (DM) patients with acute coronary syndrome (ACS) undergoing Percutaneous Coronary Intervention (PCI) by tertiles of HbA1c content.

## Abbreviations

BMI: body mass index
Prior MI: prior myocardial infarction
Prior PCI: prior percutaneous coronary intervention
SBP: systolic blood pressure
DBP: diastolic blood pressure
eGFR: estimated glomerular filtration rate
FBS: fasting blood sugar
TC: total cholesterol
TG: triglycerides
HDL-C: high-density lipoprotein cholesterol
LDL-C: low-density lipoprotein cholesterol
Apo-A1: apolipoprotein A1
Apo-B: apolipoprotein B
NT-proBNP: N-terminal pro-B-type natriuretic peptide
PLT: platelets
LVEF: left ventricle ejection fraction
WBC: white blood cell count
ACEI: angiotensin-converting enzyme inhibition
ARB: angiotensin receptor blocker
ANOVA: one-way analysis of variance
HbA1c: HemoglobinA1c
MACEs: major adverse cardiac events
DM: diabetes mellitus
ACS: acute coronary syndrome
AUC: the receiver-operating characteristic curve
ROC: receiver-operating characteristic
GRACE: the Global Registry of Acute Coronary Events
NSTEMI: non-ST-segment elevation myocardial infarction
STEMI: ST-segment elevation MI
IDI: integrated discrimination improvement
NRI: net reclassification improvement
