# Supplementary material for: Adjustment of the GRACE score by HemoglobinA1c enables a more accurate prediction of long-term major adverse cardiac events in acute coronary syndrome without diabetes undergoing percutaneous coronary intervention
Source: Cardiovasc Diabetol. 2015 Aug 19;14:110. doi: 10.1186/s12933-015-0274-4 (PMC4541750; doi:10.1186/s12933-015-0274-4)
Supplement: Additional file 1: — Table S1. Baseline characteristics of 533 non-diabetes mellitus (DM) patients with acute coronary syndrome (ACS) undergoing Percutaneous Coronary Intervention (PCI) by tertiles of HbA1c content. [file 12933_2015_274_MOESM1_ESM.docx]

| Variable | Tertile 1  ≤5.4%  (n=192) | Tertile 2  5.5–5.8%  (n=171) | Tertile 3 5.9–6.4%  (n=170) | *p* value |
| --- | --- | --- | --- | --- |
| Age, year | 58.38±13.32 | 60.39±13.07 | 61.31±11.26 | 0.076 |
| Sex |  |  |  |  |
| Male | 142 (74.0) | 112 (65.5) | 107 (62.9) | 0.061 |
| BMI, kg/m^2^ | 24.24±2.61 | 23.85±2.91 | 24.24±2.57 | 0.298 |
| Hypertension | 67 (34.9) | 64 (37.4) | 64 (37.6) | 0.831 |
| Smoking | 122 (63.5) | 98(57.3) | 113 (66.5) | 0.202 |
| Prior MI | 13 (6.8) | 11 (6.4) | 13 (7.6) | 0.901 |
| Prior PCI | 8 (4.2) | 3 (1.8) | 13 (7.6) | **0.031** |
| DBP, mmHg | 78.20±13.22 | 77.62±12.88 | 77.98±12.97 | 0.913 |
| SBP, mmHg | 125.93±20.92 | 125.73±19.89 | 124.88±19.20 | 0.872 |
| Heart rate, bpm | 75.32±13.97 | 75.22±13.15 | 75.55±12.65 | 0.972 |
| eGFR, ml·min^–1^·1.73 m^–2^ | 95.57±40.63 | 84.40±31.19 | 84.95±32.19 | **0.003** |
| FBS, mmol/L | 6.16±1.48 | 6.419±1.81 | 6.57±2.13 | 0.095 |
| TC, mmol/L | 3.81±0.82 | 3.91±0.97 | 4.07±1.39 | 0.066 |
| TG, mmol/L | 1.66±0.88 | 1.62±1.23 | 1.58±0.88 | 0.775 |
| HDL, mmol/L | 1.01±0.24 | 1.03±0.25 | 1.03±0.25 | 0.569 |
| LDL, mmol/L | 2.24±0.65 | 2.28±0.79 | 2.31±0.79 | 0.629 |
| Apo A1, g/L | 1.08±0.18 | 1.08±0.16 | 1.09±0.21 | 0.772 |
| Apo B, g/L | 0.75±0.21 | 0.75±0.23 | 0.79±0.24 | 0.176 |
| LVEF (%) | 55.53±12.17 | 54.68±11.72 | 52.39±12.98 | **0.047** |
| LnNT-proBNP, | 6.02±1.45 | 5.45±1.64 | 6.08±1.66 | **＜0.001** |
| PLT count, 10^9^/L | 192.61±71.58 | 184.56±52.85 | 201.33±76.73 | 0.076 |
| WBC count, 10^9^/L | 7.84±3.17 | 7.96±3.17 | 8.38±3.35 | 0.259 |
| Monocyte count, 10^9^/L | 0.53±0.26 | 0.56±0.35 | 0.62±0.36 | **0.028** |
| Neutrophile count, 10^9^/L | 5.88±3.17 | 5.82±3.06 | 6.06±3.19 | 0.752 |
| Madication at discharge | | | | |
| Aspirin | 189 (98.4) | 169 (98.8) | 166 (97.65) | 0.688 |
| Clopidogrel | 190(99.0) | 167(97.7) | 170(100) | 0.122 |
| Statins | 152 (79.2) | 135 (78.9) | 143 (84.1) | 0.387 |
| ACEI/ARB | 146(76.0) | 116 (67.8) | 127 (74.7) | 0.177 |
| β-blocker | 133 (69.3) | 1131 (66.1) | 101 (59.4) | 0.138 |
